# Targeting ERK enhances the cytotoxic effect of the novel PI3K and mTOR dual inhibitor VS-5584 in preclinical models of pancreatic cancer

**DOI:** 10.18632/oncotarget.17869

**Published:** 2017-05-15

**Authors:** Changwen Ning, Min Liang, Shuang Liu, Guan Wang, Holly Edwards, Yang Xia, Lisa Polin, Gregory Dyson, Jeffrey W. Taub, Ramzi M. Mohammad, Asfar S. Azmi, Lijing Zhao, Yubin Ge

**Affiliations:** ^1^ National Engineering Laboratory for AIDS Vaccine, Key Laboratory for Molecular Enzymology and Engineering of the Ministry of Education, School of Life Sciences, Jilin University, Changchun, P.R. China; ^2^ Department of Oncology, Wayne State University School of Medicine, Detroit, MI, USA; ^3^ Molecular Therapeutics Program, Barbara Ann Karmanos Cancer Institute, Wayne State University School of Medicine, Detroit, MI, USA; ^4^ Department of Pathology, The Second Hospital of Jilin University, Changchun, P.R. China; ^5^ Department of Pediatrics, Wayne State University School of Medicine, Detroit, MI, USA; ^6^ Division of Pediatric Hematology/Oncology, Children's Hospital of Michigan, Detroit, MI, USA; ^7^ Department of Rehabilitation, School of Nursing, Jilin University, Changchun, P.R. China

**Keywords:** pancreatic cancer, PI3K/mTOR, ERK, VS-5584, SCH772984

## Abstract

Pancreatic ductal adenocarcinoma (PDAC) is a deadly disease in urgent need of newer therapeutic modalities. Majority of patients with PDAC have mutations in KRAS, which unfortunately remains an ineffectual target. Our strategy here is to target KRAS downstream effectors PI3K and mTOR. In this study, we investigated the antitumor efficacy of the novel PI3K and mTOR dual inhibitor VS-5584 in PDAC. Our data shows that PI3K/mTOR dual inhibition causes ERK activation in all tested PDAC cell lines. Although the MEK inhibitor GSK1120212 could abrogate VS-5584-induced ERK activation, it did not substantially enhance cell death in all the cell lines tested. However, combination with ERK inhibitor SCH772984 not only mitigated VS-5584-induced ERK activation but also enhanced VS-5584-induced cell death. In a xenograft model of PDAC, we observed 28% and 44% tumor inhibition for individual treatment with VS-5584 and SCH772984, respectively, while the combined treatment showed superior tumor inhibition (80%) compared to vehicle control treatment. Our findings support the clinical development of VS-5584 and ERK inhibitor combination for PDAC treatment.

## INTRODUCTION

Pancreatic cancer has a 5-year survival rate of only 8% in the United States [1]. Gemcitabine has been the gold standard for treating advanced unresectable pancreatic cancer since the US FDA approved its use in 1996, yet it only offers a modest benefit of 4.6 months survival [2, 3]. Recently, nab-paclitaxel was shown to improve gemcitabine efficacy, resulting in a median overall survival rate of 8.5 months [4]. It has been predicted that, by the year 2020, pancreatic cancer will be the second-leading cause of cancer-related death in the United States [5], highlighting the urgency for new treatment options for this deadly disease.

Pancreatic ductal adenocarcinoma (PDAC) is the most common type of pancreatic cancer, accounting for about 90% of pancreatic cancers. Activating KRAS mutations occur in over 90% of PDAC cases [6, 7] and drive PDAC cell proliferation and survival. Inhibition of mutant KRAS has proven to be a difficult task, thus alternative therapies targeting downstream KRAS effectors have been exploited [6, 8]. The PI3K/mTOR pathway, which is downstream of KRAS, is commonly deregulated in many cancer types [9]. The PI3K/mTOR pathway plays a key role in proliferation, migration, survival, and growth [10]. The first inhibitors of the PI3K/mTOR signaling pathways were rapamycin and rapalogs, which targeted mTOR. Unfortunately, rapalogs have demonstrated limited clinical benefits due to drug-induced feedback loops, which cause hyperactivation of PI3K/AKT and enhance the proliferation rate of tumors [11, 12]. These findings have led to the development of dual PI3K and mTOR inhibitors which can overcome feedback activation of PI3K resulting from mTOR inhibition. However, efficacy can be hindered by activation of other pathways.

The MEK/ERK pathway is another downstream pathway affected by KRAS. Cross-talk between the PI3K/mTOR and the MEK/ERK signaling pathways has been reported (Supplementary Figure 1) [13–18]. MEK inhibition has been shown to cause PI3K activation and PI3K inhibition has been demonstrated to cause ERK activation [11, 19, 20]. Thus, targeting both pathways simultaneously may be a promising approach to treat PDAC.

In this study, we investigated the novel dual PI3K/mTOR inhibitor VS-5584 in PDAC cell lines. We found that VS-5584 treatment causes activation of ERK and that targeting MEK, which is upstream of ERK, does not enhance VS-5584 antitumor activity in a wild-type *KRAS* PDAC cell line. We also found that the ERK-selective inhibitor SCH772984 enhances the antitumor activity of VS-5584 resulting in significant enhancement of cell death and significant inhibition of cell migration in a wild-type and a mutant *KRAS* PDAC cell line. Furthermore, our *in vivo* studies revealed that the combined drug treatment significantly inhibited tumor growth in a PDAC xenograft mouse model. Our studies provide support for the clinical development of combined VS-5584 and an ERK inhibitor for the treatment of pancreatic cancer.

## RESULTS

### VS-5584 treatment results in inactivation of PI3K and mTOR, but activation of ERK in PDAC cell lines

**Figure 1:**
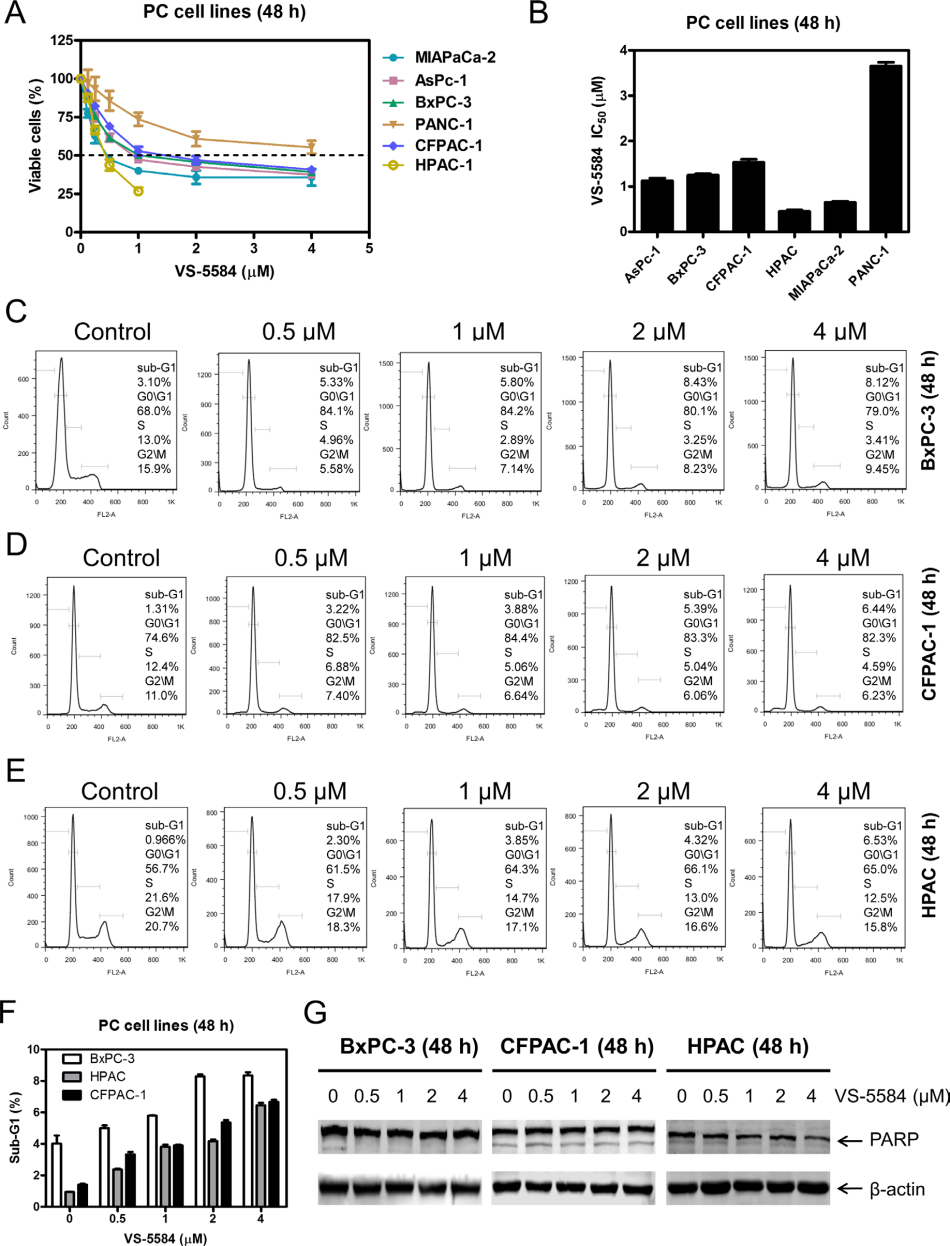
VS-5584 treatment decreases the percentage of S and G2/M phase cells and induces minimal cell death in PDAC cell lines (**A**) PDAC cell lines were treated with vehicle control or variable concentrations of VS-5584 in 96-well plates for 48 h and viable cells were determined using MTT assays. (**B**) IC_50_ values were calculated as drug concentration necessary to inhibit 50% OD_590_ compared to vehicle control treated cells. Data are graphed as mean ± SEM from three independent experiments. (**C**–**E**) BxPC-3, CFPAC-1, and HPAC cells were treated with vehicle control or variable concentrations of VS-5584 for 48 h, then fixed with 80% ice-cold ethanol and stained with PI for cell cycle analysis. Representative histograms are shown. (**F**) The sub-G1 data are presented as means of triplicates ± SEM from one representative experiment. (**G**) BxPC-3, CFPAC-1, and HPAC cells were treated with vehicle control or the indicated concentrations of VS-5584 for 48 h. Whole cell lysates were subjected to Western blotting and probed with anti-PARP or -β-actin antibody.

First, we used MTT assays to determine VS-5584 sensitivities in 6 PDAC cell lines. VS-5584 IC_50_s were variable, ranging from about 0.45 to 3.7 μM (Figure 1A and 1B). Next, we treated PDAC cell lines with 0–4 μM VS-5584 for 48 h, fixed the cells in ethanol, and then subjected them to PI staining and flow cytometry analyses. In BxPC-3, CFPAC-1, and HPAC cells, VS-5584 treatment decreased the percentage of cells in the S and G2/M cell cycle phases and increased the percentage of G0/G1 cells (Figure 1C–1E). VS-5584 did not induce appreciable levels of cell death, as assessed by sub-G1 analysis and PARP cleavage (Figure 1F and 1G).

**Figure 2:**
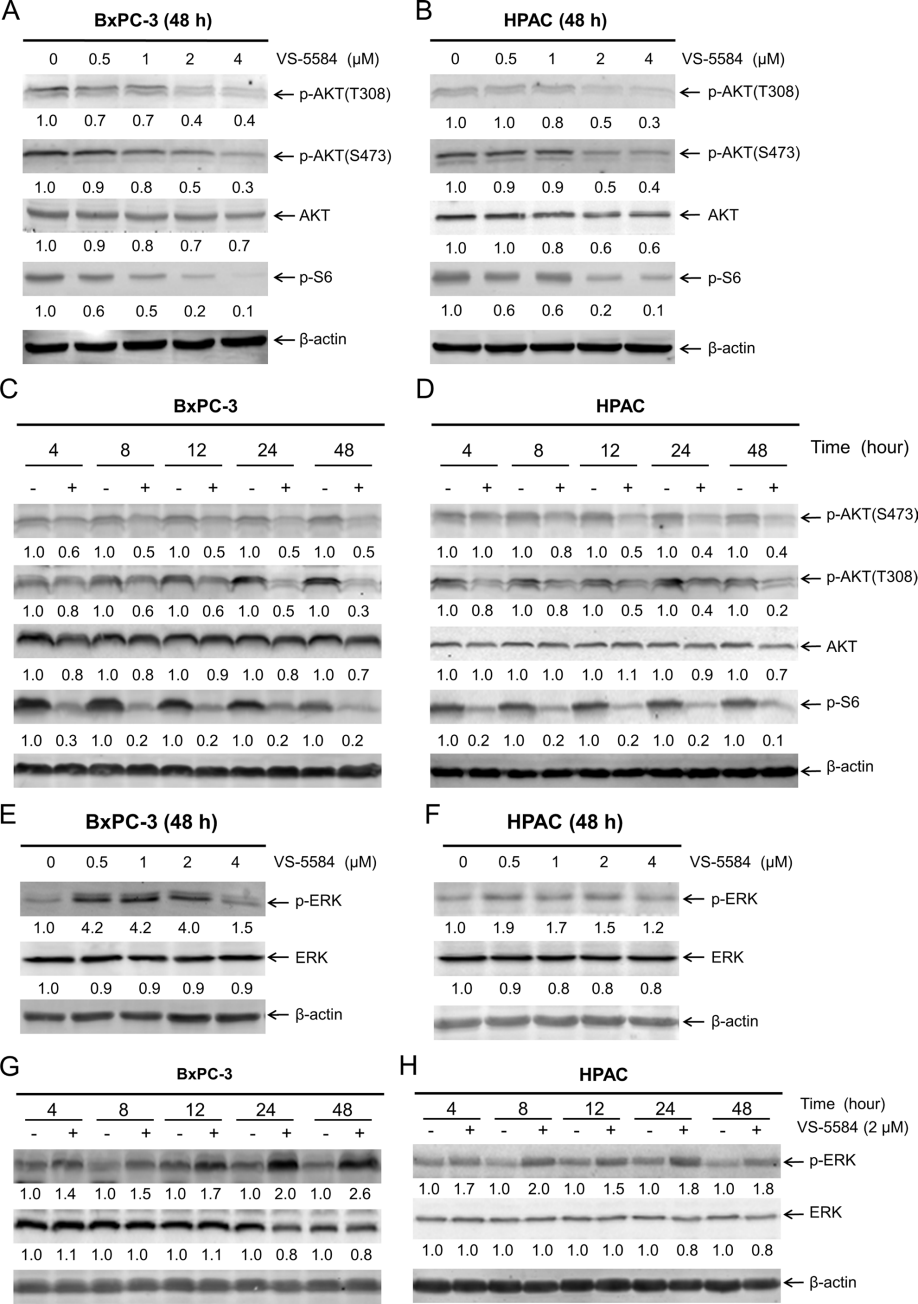
VS-5584 treatment causes activation of ERK in PDAC cells (**A** and **B**) BxPC-3 and HPAC cells were treated with vehicle control or variable concentrations of VS-5584 for 48 h. Whole cell lysates were subjected to Western blotting and probed with the indicated antibody. The fold changes for the densitometry measurements, normalized to β-actin and then compared to vehicle control, are indicated. (**C** and **D**) BxPC-3 and HPAC cells were treated with vehicle control or 2 μM VS-5584 for 4, 8, 12, 24, or 48 h. Whole cell lysates were subjected to Western blotting and probed with anti-p-AKT(S473), -p-AKT(T308), -AKT, -p-S6, or -β-actin antibody. The fold changes for the densitometry measurements, normalized to β-actin and then compared to no drug treatment control, are indicated. (**E** and **F**) BxPC-3 and HPAC cells were treated with vehicle control or variable concentrations of VS-5584 for 48 h. Whole cell lysates were subjected to Western blotting and probed with anti-p-ERK, -ERK, or -β-actin antibody. The fold changes for the densitometry measurements, normalized to β-actin and then compared to no drug treatment control, are indicated. (**G** and **H**) BxPC-3 and HPAC cells were treated with vehicle control or 2 μM VS-5584 for 4, 8, 12, 24, or 48 h. Whole cell lysates were subjected to Western blotting and probed with anti-p-ERK, -ERK, or -β-actin antibody. The fold changes for the densitometry measurements, normalized to β-actin and then compared to no drug treatment control, are indicated.

To confirm that VS-5584 inhibits both PI3K and mTOR, we treated BxPC-3 and HPAC cells with variable concentrations of VS-5584 for 48 h. Western blotting revealed that VS-5584 inhibited both PI3K and mTOR as demonstrated by a concentration-dependent decrease of p-AKT(T308), p-AKT(S473), and p-S6 (Figure 2A and 2B). p-S6 was markedly decreased after treatment with 0.5 μM VS-5584 in both cell lines, while substantial decrease of p-AKT(T308) and p-AKT(S473) occurred at concentrations of 2 μM and higher. In BxPC-3 cells, time course experiments revealed noticeably decreased p-S6 and p-AKT(S473) as early as 4 h following treatment, while markedly decreased p-AKT(T308) was not detected until 8 h after treatment (Figure 2C). In HPAC cells, 2 μM VS-5584 caused substantial decrease of p-S6 by 4 h post-treatment, while decreased p-AKT(S473) and p-AKT(T308) were not detected until 12 h post-VS-5584 treatment (Figure 2D). Despite inhibition of both PI3K and mTOR, VS-5584 did not induce an appreciable amount of cell death (Figure 1F). These results suggest that VS-5584 treatment may have activated another cell survival pathway which prevented cell death. It has been reported that mTOR inhibition can lead to overactivation of the MEK/ERK pathway [21, 22]. To determine if this happens in PDAC cells, we treated BxPC-3 and HPAC cells with variable concentrations of VS-5584 and subjected whole cell lysates to Western blot analysis. The blots revealed considerable increase of p-ERK at concentrations as low as 0.5 μM (Figure 2E and 2F). Time course experiments showed increased phosphorylation of ERK 4 h after VS-5584 treatment (Figure 2G and 2H). Taken together, these results suggest that activation of the MEK/ERK pathway may mediate resistance to VS-5584 in PDAC cells.

### Inhibition of MEK abrogates VS-5584-induced increase of phosphorylated ERK and substantially enhances VS-5584-induced cell death in HPAC cells

**Figure 3:**
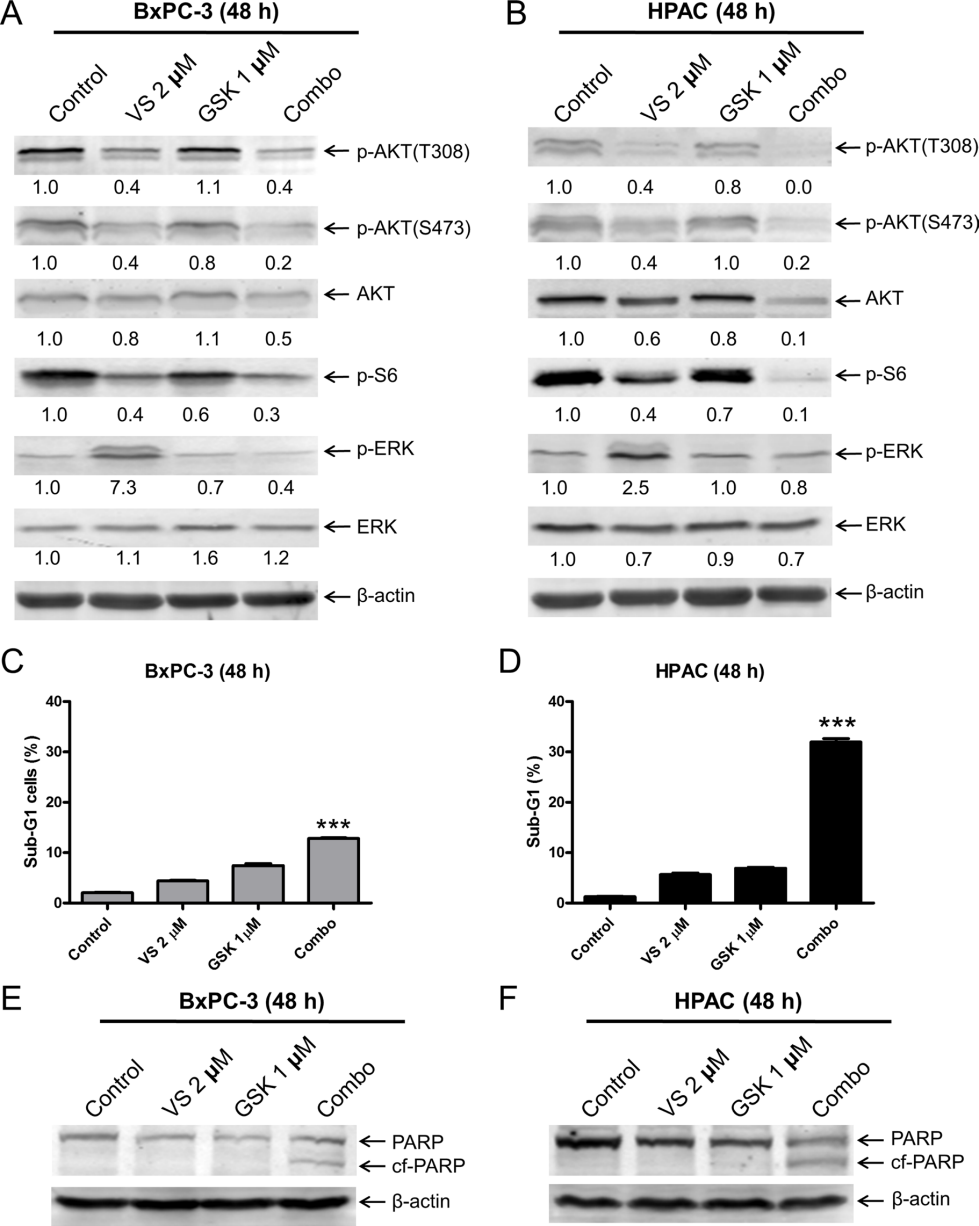
Inhibition of MEK abrogates VS-5584-induced phosphorylation of ERK, but substantially enhances VS-5584-induced cell death in a cell line-selective fashion (**A** and **B**) BxPC-3 and HPAC cells were treated with vehicle control, 2 μM VS-5584 (VS), 1 μM GSK1120212 (GSK), or 2 μM VS plus 1 μM GSK (Combo) for 48 h. Whole cell lysates were subjected to Western blotting and probed with the indicated antibody. The fold changes for the densitometry measurements, normalized to β-actin and then compared to no drug treatment control, are indicated. (**C** and **D**) BxPC-3 and HPAC cells were treated with vehicle control or the indicated drugs for 48 h. Cells were fixed with 80% ice-cold ethanol and stained with PI for cell cycle analysis. The percentage of cells with sub-G1 DNA content are graphed as means of triplicates ± SEM from one representative experiment. ***indicates *p* < 0.001; combined treatment compared to individual treatments and control. (**E** and **F**) BxPC-3 and HPAC cells were treated with vehicle control or the indicated drugs for 48 h. Whole cell lysates were subjected to Western blotting and probed with anti-PARP or -β-actin antibody.

To determine if MEK inhibition prevents VS-5584-induced ERK activation, we treated PDAC cell lines BxPC-3 and HPAC with VS-5584 and the MEK inhibitor GSK1120212 (approved by the US FDA for the treatment of metastatic melanoma) alone or in combination. GSK1120212 treatment decreased p-ERK in BxPC-3 cells, while levels remained unchanged in HPAC cells. However, when combined with VS-5584, GSK1120212 abrogated ERK activation induced by VS-5584 in both cell lines (Figure 3A and 3B). However, in HPAC cells VS-5584 treatment caused a small decrease in total ERK levels which was maintained in the combined drug treatment. Similar to VS-5584, GSK1120212 treatment alone did not induce robust cell death either (Figure 3C). However, it caused significantly enhanced VS-5584-induced cell death in HPAC cells (approximately 30%, *p* < 0.001 combination compared to control, VS, or GSK, Figure 3D). In contrast, its enhancement on VS-5584-induced cell death in BxPC-3 cells was minimal, even though statistically significant (< 15% sub-G1, *p* < 0.001 combination compared to control, VS, or GSK, Figure 3C–3F). These results suggest that MEK inhibition can overcome VS-5584-induced ERK activation in PDAC cells. However, robust induction of cell death by combined VS-5584 and GSK1120212 appears to be selective.

### ERK inhibition overcomes resistance to VS-5584 in PDAC cells regardless of KRAS status

**Figure 4:**
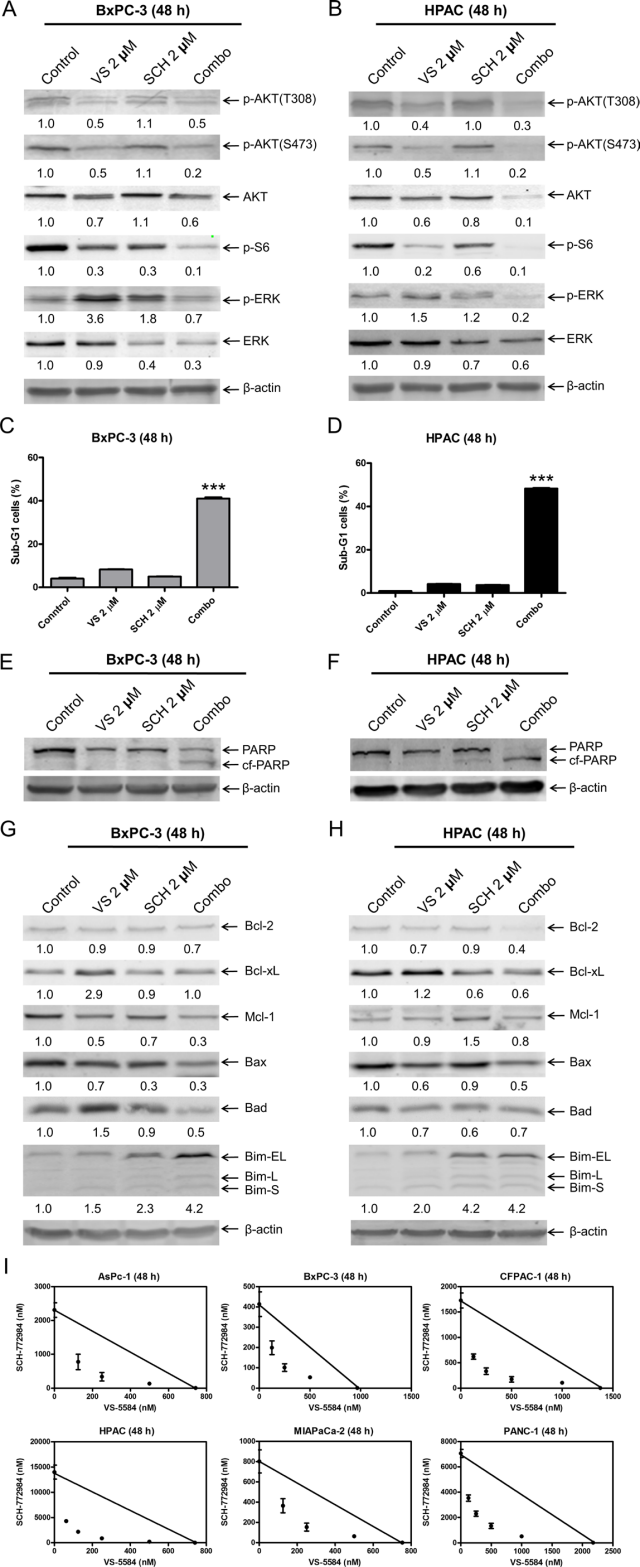
ERK inhibition overcomes resistance to VS-5584 in PDAC cells (**A** and **B**) BxPC-3 and HPAC cells were treated with vehicle control, 2 μM VS, 2 μM SCH772984 (SCH), or 2 μM VS plus 2 μM SCH (Combo) for 48 h. Whole cell lysates were subjected to Western blotting and probed with the indicated antibody. (**C** and **D**) BxPC-3 and HPAC cells were treated with vehicle control, 2 μM VS, 2 μM SCH, or 2 μM VS plus 2 μM SCH for 48 h. Cells were fixed with 80% ice-cold ethanol and stained with PI for cell cycle analysis. The percentage of cells with sub-G1 DNA content are graphed as means of triplicates ± SEM from one representative experiment. ***indicates *p* < 0.001; combined treatment compared to individual treatments and control. (**E**–**H**) BxPC-3 and HPAC cells were treated with vehicle control or the indicated drugs for 48 h. Whole cell lysates were subjected to Western blotting and probed with the indicated antibody. The fold changes for the densitometry measurements, normalized to β-actin and then compared to no drug treatment control, are indicated. (**I**) AsPC-1, BxPC-3, CFPAC-1, HPAC, MIAPaCa-2, and PANC-1 cells were treated with vehicle control or variable concentrations of SCH and VS-5584, alone or in combination, for 48 h. Viable cells were measured by MTT assays. Standard isobologram analyses of the antitumor interactions are shown. The IC_50_ values of each drug are plotted on the axes; the solid line represents the additive effect, while the points represent the concentrations of each drug resulting in 50% inhibition of proliferation. Points falling below the line indicate synergism whereas those above the line indicate antagonism.

Since VS-5584 treatment causes increased activation of ERK, we investigated the effects of ERK inhibition in combination with VS-5584. We treated PDAC cells with VS-5584 and the ERK-selective inhibitor SCH772984, alone or in combination, for 48 h. SCH772984 treatment caused decrease of p-S6 and total ERK. Similar decrease was also observed in the combined drug treatment. VS-5584 treatment caused an increase in p-ERK, which was substantially decreased by combined drug treatment. In the combined drug treatment, p-ERK and p-AKT (S473) levels were decreased compared to individual treatment (Figure 4A and 4B). Combined VS-5584 and SCH772984 treatment caused significant induction of cell death, as determined by PI staining and sub-G1 analysis (*p* < 0.001, combination compared to control or individual drug treatment), and detection of cleaved PARP (Figure 4C–4F).

It has been reported that both PI3K/mTOR and MEK/ERK signaling pathways regulate the protein levels of the Bcl-2 family [23–25]. It is conceivable that VS-5584 and SCH772984 cooperate in regulating Bcl-2 family proteins to induce cell death in PDAC cells. To test this possibility, the effects of VS-5584 and SCH772984, alone or in combination, on the protein levels of the Bcl-2 family members were investigated in BxPC-3 and HPAC cell lines. VS-5584 treatment increased Bcl-xL and Bim protein levels, and decreased Bax protein levels (Figure 4G and 4H). Bim expression was increased following SCH772984 treatment and remained increased in the combined treatment in both cell lines. Although SCH772984 treatment had little to no effect on Bcl-xL levels, it completely abolished VS-5584-induced Bcl-xL expression in BxPC-3 cells. Similar results were also obtained in HPAC cells, except SCH772984 treatment caused an obvious decrease of Bcl-xL levels. Although both SCH772984 and VS-5584 alone had minimal effect on Bcl-2 levels in BxPC-3 cells, combined treatment resulted in decrease of Bcl-2. Similar results were also obtained in HPAC cells, except VS-5584 treatment resulted in a bigger decrease of Bcl-2. Mcl-1 levels were decreased in BxPC-3 cells after treatment with the drugs, both individually and combined. In HPAC cells, VS-5584 treatment had no effect on expression of Mcl-1, while there was an increase after SCH772984 treatment, which was abrogated by the combined drug treatment. Although VS-5584 treatment caused an increase of Bad in BxPC-3 cells, it was abolished by the addition of SCH772984. In contrast to BxPC-3 cells, the drug treatments caused decrease of Bad in HPAC cells. These results demonstrate that VS-5584 and SCH772984, alone or in combination, have wide effects on the expression levels of Bcl-2 family proteins and suggest that the net effect of the changes in expression of Bcl-2 family proteins favor cell death.

**Figure 5:**
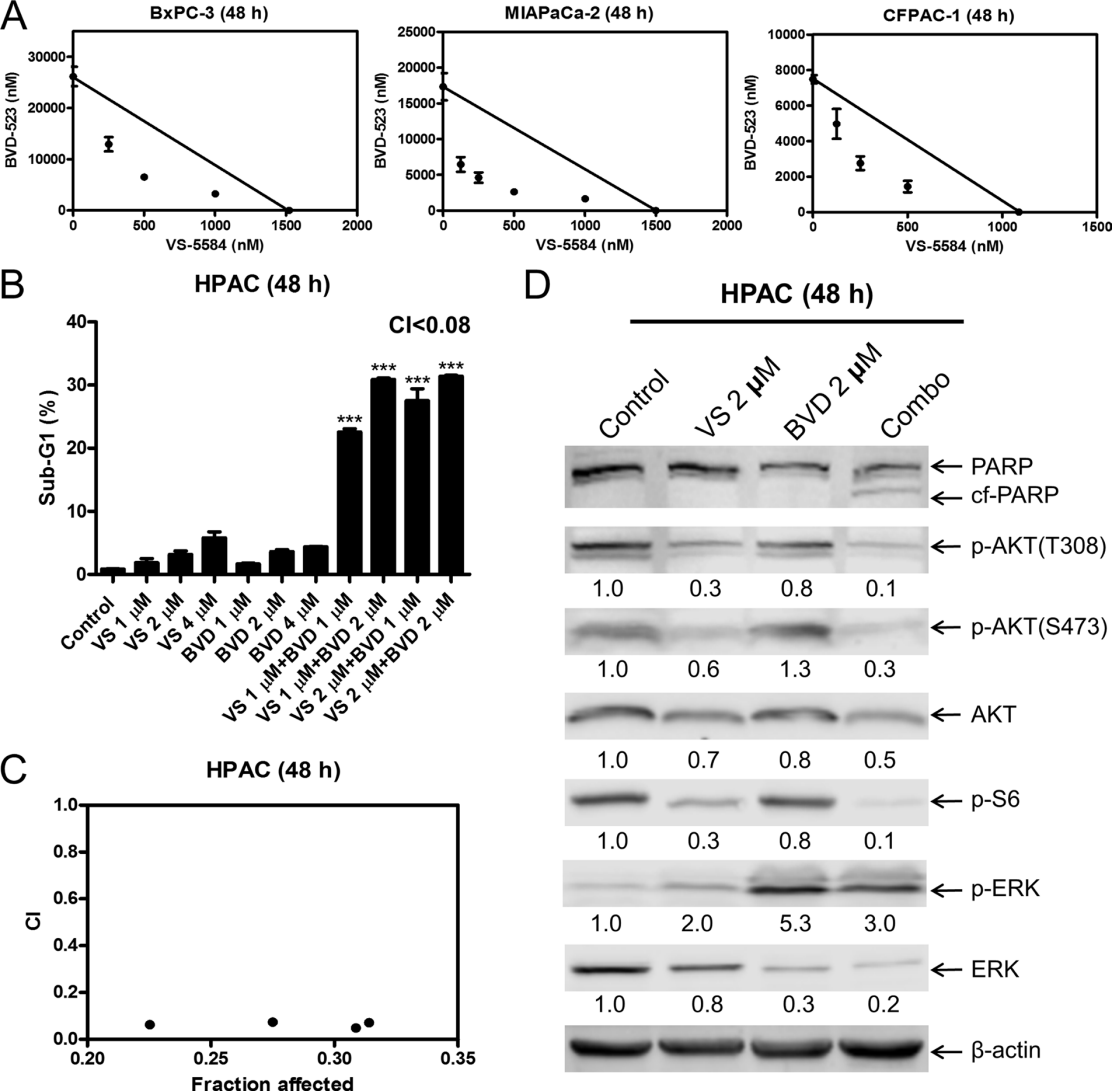
BVD-523 synergizes with VS-5584 in AML cells (**A**) BxPC-3, MIAPaCa-2, and CFPAC-1 cells were treated with vehicle control or variable concentrations of BVD-523 and VS-5584, alone or in combination, for 48 h. Viable cells were measured by MTT assays. Standard isobologram analyses of the antitumor interactions are shown. The IC_50_ values of each drug are plotted on the axes; the solid line represents the additive effect, while the points represent the concentrations of each drug resulting in 50% inhibition of proliferation. Points falling below the line indicate synergism whereas those above the line indicate antagonism. (**B**) HPAC cells were treated with vehicle control, VS, BVD-523 (BVD), or VS plus BVD for 48 h. Cells were fixed with 80% ice-cold ethanol and stained with PI for cell cycle analysis. The percentage of cells with sub-G1 DNA content are graphed as means of triplicates ± SEM from one representative experiment. ***indicates *p* < 0.001; combined treatment compared to individual treatments and vehicle control. Combination index (CI) values were calculated using CompuSyn software. (**C**) CI vs. Fa plot (combination index vs. fraction affected) for the cell death data is presented. (**D**) HPAC cells were treated with vehicle control or the indicated drugs for 48 h. Whole cell lysates were subjected to Western blotting and probed with the indicated antibody. The fold changes for the densitometry measurements, normalized to β-actin and then compared to no drug treatment control, are indicated.

To determine the extent and direction of antitumor interactions between VS-5584 and SCH772984, we performed MTT assays and standard isobologram analyses with 6 PDAC cell lines. Synergistic antitumor interactions of VS-5584 and SCH772984 were detected in all the cell lines tested (*n* = 6; Figure 4I). To rule out off-target effects, we performed MTT assays in PDAC cell lines BxPC-3, MIAPaCa-2, and CFPAC-1, using VS-5584 and BVD-523, an ERK-selective inhibitor structurally-unrelated to SCH772984. Synergistic antitumor interactions between VS-5584 and BVD-523 were detected in the PDAC cell lines tested (*n* = 3), as determined by MTT assays and standard isobologram analyses (Figure 5A). In HPAC cells, the IC_50_ for BVD-523 was not reached using concentrations up to 32 μM. Thus, synergy in HPAC cells was determined by flow cytometry measurement of cell death and calculation of the combination index values. Consistent with the results obtained with SCH772984, combined VS-5584 and BVD-523 treatment caused significant induction of cell death in HPAC cells (*p* < 0.001, compared to control, VS-5584, or SCH772984; Figure 5B). Combination induced cell death was determined to be synergistic in HPAC cells (CI<0.08, Figure 5B and 5C). Western blots confirmed inhibition of AKT and S6 by the drug treatments in HPAC cells (Figure 5D). Surprisingly, BVD-523 treatment caused dramatic increase of ERK phosphorylation and did not abolish VS-5584-induced ERK phosphorylation. However, decreased total ERK was detected in HPAC cells following BVD-523 treatment, which was maintained in the combined treatment. These results further confirm that inhibition of ERK and/or down-regulation of ERK rather than abolishment of its phosphorylation abrogates resistance to VS-5584 in PDAC cells.

### VS-5584 combined with SCH772984 reduces PDAC cell migration

**Figure 6:**
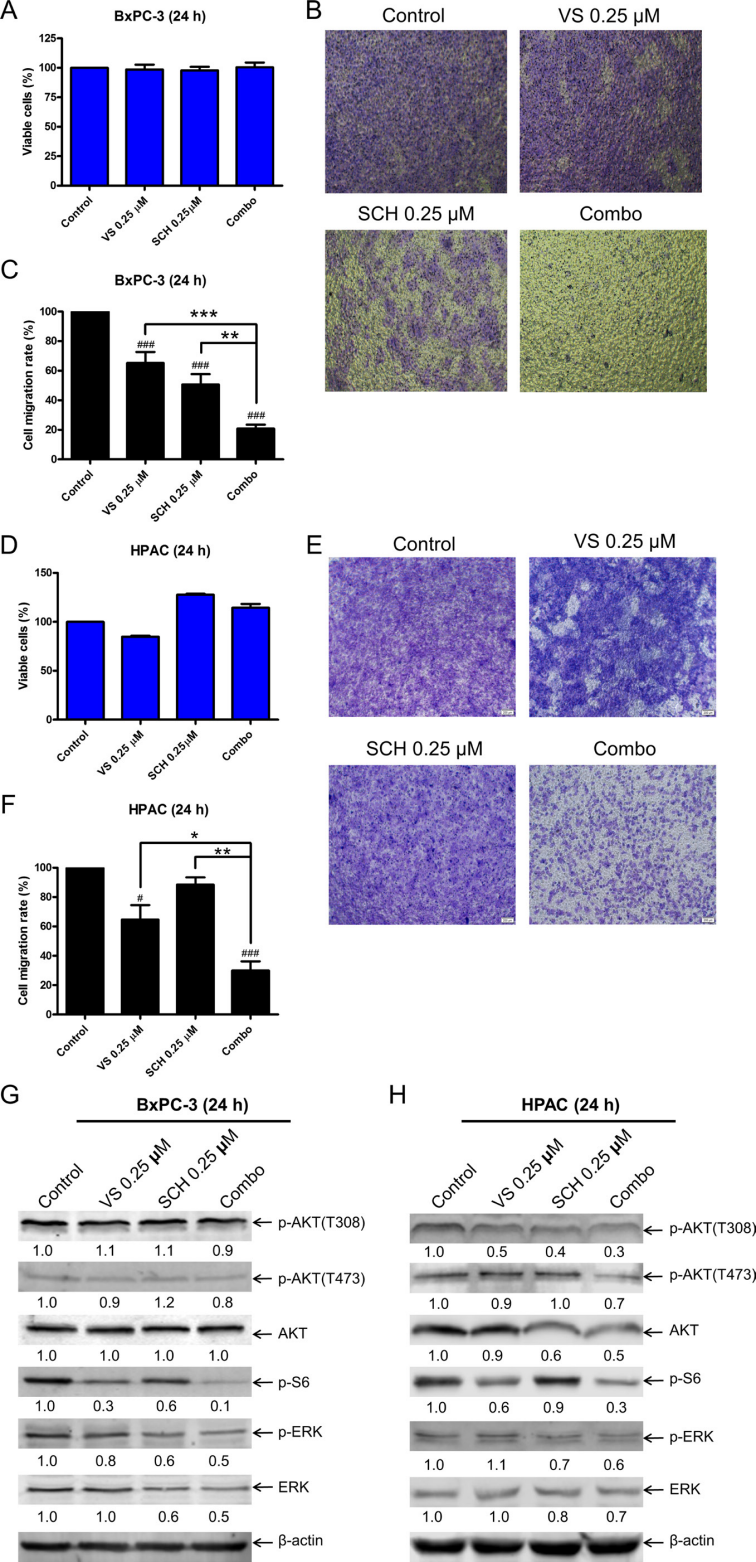
Combined ERK inhibition and VS-5584 treatment decreases migration of PDAC cell lines (**A**) BxPC-3 cells were treated with vehicle control or VS-5584 and SCH772984, alone or in combination, for 24 h. Viable cells were determined by MTT assays. Results are graphed as mean ± SEM from 3 independent experiments. (**B**) Transwell migration assays were performed as described in the ‘Materials and methods’ section. Representative images are shown. (**C**) Results were obtained from three independent transwell migration experiments. The cell migration rates, compared to vehicle control, are shown as mean ± SEM. **indicates *p* < 0.01 and ***indicates *p* < 0.001; combined treatment compared to individual drug treatments. ^###^indicates *p* < 0.001; indicated treatment compared to control. (**D**) HPAC cells were treated with vehicle control or VS-5584 and SCH772984, alone or in combination, for 24 h. Viable cells were determined by MTT assays. Results are graphed as mean ± SEM. (**E**) Transwell migration assays were performed as described in the ‘Materials and methods’ section. Representative images are shown. (**F**) Results were obtained from three independent transwell experiments. The cell migration rates, compared to control, are shown as mean ± SEM. *indicates *p* < 0.05 and **indicates *p* < 0.01; combined treatment compared to individual drug treatments. ^#^indicates *p* < 0.05 and ^###^indicates *p* < 0.001; indicated treatment compared to control. (**G** and **H**) BxPC-3 and HPAC cells were treated with vehicle control, 0.25 μM VS, 0.25 μM SCH, or in combination for 24 h. Whole cell lysates were subjected to Western blotting and probed with the indicated antibody. The fold changes for the densitometry measurements, normalized to β-actin and then compared to no drug treatment control, are indicated.

In order to determine if VS-5584 and SCH772984 affect pancreatic cancer cell migration, we treated BxPC-3 cells with VS-5584 and SCH772984, alone or in combination for 24 h, with concentrations of drug which did not have a significant impact on the percent of viable cells (Figure 6A). The cells were then plated in transwell chambers. Both VS-5584 and SCH772984 treatment decreased the cell migration rate (control versus VS, *p* < 0.001; control versus SCH, *p* < 0.001) while the combination significantly decreased the cell migration rate compared to vehicle control (*p* < 0.001) and individual drug treated cells (combination versus VS, *p* < 0.001; combination versus SCH, *p* < 0.01; Figure 6B and 6C). HPAC cells were treated for 24 h with the same concentrations of VS-5584 and SCH772984 as BxPC3 cells. Although VS-5584 treatment did cause a small decrease of viable cells, SCH772984 and combined treatment both caused a small increase in viable cells (Figure 6D). Similar to the BxPC-3 cells, combination treatment in HPAC cells caused significant decrease of cell migration compared to vehicle control and individual drug treatments (*p* < 0.05, Figure 6E–6F). To determine the biological effects of VS-5584 and SCH772984 at the lower concentrations used in the cell migration assays, BxPC-3 and HPAC cells were treated with 0.25 μM VS-5584 and 0.25 μM SCH772984 for 24 h, alone or in combination, and then whole cell lysates were subjected to Western blot analysis. BxPC-3 and HPAC cells treated with VS-5584 alone had decreased levels of p-S6, while SCH772984 treatment decreased p-ERK levels in both cell lines (Figure 6G and 6H). In the combined treatment p-S6 was further decreased compared to individual drug treatment in both cell lines. p-ERK levels were reduced to levels similar to SCH772984 treatment alone. Both p-AKT(S473) and p-AKT(T308) levels were minimally affected by the individual and combined treatments. Total ERK levels decreased in BxPC-3 cells treated with SCH772984. The combined drug treatment caused decrease of total ERK levels compared to control and single drug treatment in both cell lines. These results suggest that the combined treatment has an impact on PDAC cell migration through inhibition of mTOR complex 1 and ERK or decrease of total ERK.

### *In vivo* antitumor efficacy of VS-5584 and SCH772984 in an HPAC xenograft mouse model

**Figure 7:**
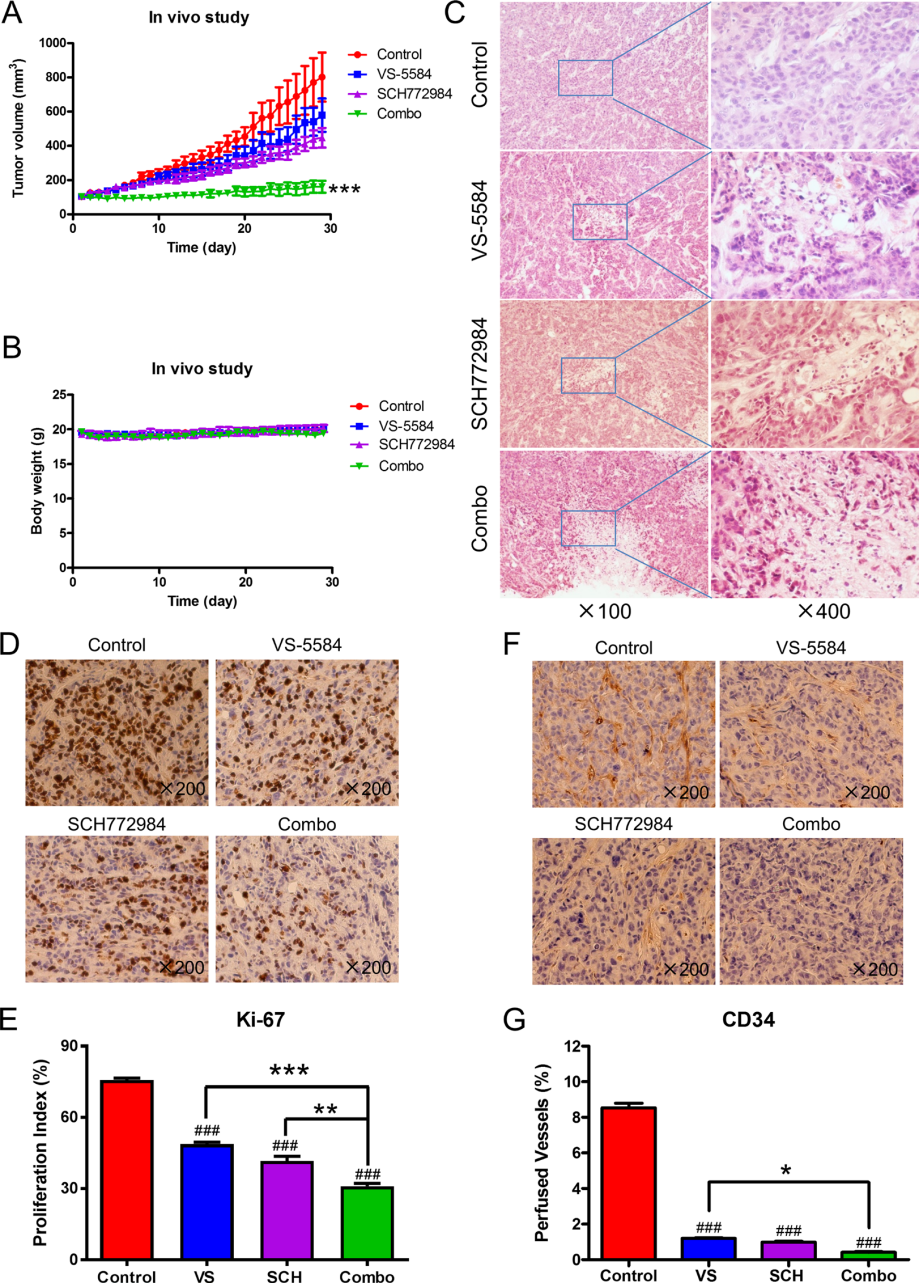
SCH772984 enhances the antitumor activity of VS-5584 in an HPAC xenograft mouse model Mice bearing HPAC xenograft tumors were treated with vehicle control, 8.4 mg/kg VS-5584, 25 mg/kg SCH772984, or the combination on a daily schedule for 4 weeks. (**A**) tumor volumes were measured daily and calculated according to the following formula: m1^2^ × m2 × 0.5236 (m1: short diameter; m2: long diameter). ***indicates *p* < 0.001. (**B**) Body weights were measured on a daily basis. (**C**–**G**) Tumor specimens (*n* = 3) obtained on day 29 were fixed in 10% formalin, embedded in paraffin, and cut into 4 μM-thick slides for H&E (panel C), Ki-67 (panel D), and CD34 staining (panel F). The proliferation index was calculated as proliferation index = Ki-67 positive cells/observed cells × 100% and graphed as means ± SEM. ** indicates *p* < 0.01 and ***indicates *p* < 0.001; combined drug treatment compared to control and individual drug treatments. ^###^indicates *p* < 0.001; indicated treatment compared to control (panel E). Perfused vessels were calculated as CD34-positive cells/observed cells × 100% and graphed as means ± SEM. *indicates *p* < 0.05; combined treatment compared to VS. ^###^indicates *p* < 0.001; indicated treatment compared to control (panel G).

Finally, we examined the *in vivo* effects of VS-5584 and SCH772984. A pilot *in vivo* study in mice was performed to determine tolerable doses for the individual drug treatments. Based on those results (data not shown), mice bearing HPAC xenograft tumors were treated daily for four weeks (QDx28) as follows: vehicle control, 8.4 mg/kg VS-5584 oral gavage (p.o.), 25 mg/kg SCH772984 intraperitoneal injection (ip), or VS-5584 and SCH772984. While individual drug treatments had an impact on tumor growth based on caliper measurements analyzed from day 29 when compared to vehicle control (72% T/C for the VS-5584 group, *p* = 0.318; 56% T/C for the SCH772984 group, *p* = 0.004, Figure 7A), the combined treatment showed significantly enhanced inhibition of tumor growth compared to vehicle control and individual drug treatments (20% T/C, *p* = 0.0006, Figure 7A). Mean starting body weights, ± SD, were 19.5 ± 1.0, 19.6 ± 0.5, 19.3 ± 1.3, and 19.7 ± 0.8 g for the vehicle control, VS-5584, SCH772984, and combination groups, respectively (Figure 7B). Over the course of the treatment period, mean body weights ranged from 19.0–20.2, 19.1–20.0, 19.0–20.2, and 18.8–19.8 g for the vehicle control, VS-5584, SCH772984, and combination groups, respectively.

**Figure 8:**
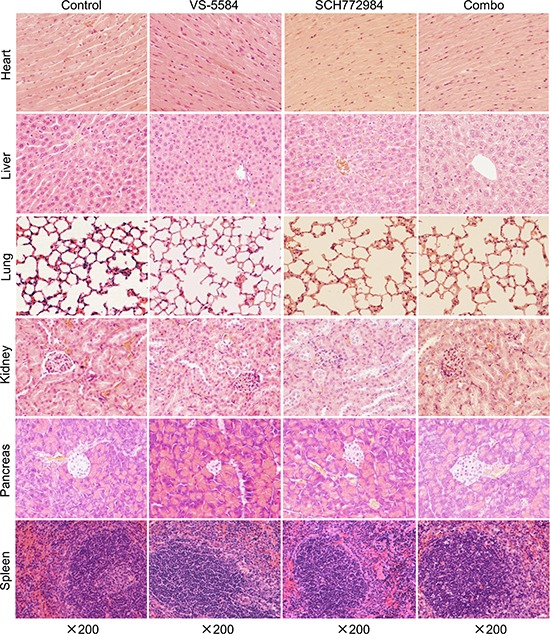
VS-5584 and SCH772984 treatment appears to be well tolerated in an HPAC xenograft mouse model Mice were sacrificed on day 29 (24 h after the last drug treatment). Major organs including heart, liver, lung, kidney, pancreas, and spleen were harvested from 3 mice in each treatment group. Tissues from these major organs were fixed in 10% formalin, embedded in paraffin, and cut into 4 μM-thick slides for H&E staining. Representative images are shown.

To further investigate the *in vivo* effects of VS-5584 and SCH772984, three tumors from each treatment arm were harvested on day 29 and analyzed by H&E and immunohistochemical staining. Individual drug treatment caused increased tumor necrosis, as indicated by H&E staining, which was further increased in the combined treatment (Figure 7C). Individual drug treatment inhibited proliferation significantly compared to vehicle control treatment (*p* < 0.001), while combination treatment significantly decreased proliferation compared to the individual drug treatment groups and the vehicle control group, as determined by Ki-67 staining and calculation of proliferation index values (*p* < 0.01, Figure 7D and 7E). There was a significant decrease of angiogenesis marker CD34 (indicator of perfused vessels) in the drug treatments compared to vehicle control (*p* < 0.001). Combined drug treatment caused a small yet significant decrease of CD34 staining compared to VS-5584 treatment (*p* < 0.05) but not to SCH772984 treatment (Figure 7F and 7G). H&E staining of tissues from heart, liver, lung, kidney, pancreas, and spleen show no appreciable difference between drug treatments and vehicle control, confirming that the drugs were well tolerated (Figure 8).

## DISCUSSION

Pancreatic cancer is a devastating disease; the 5-year survival rate is only 8% [1]. Most cases have mutationally activated KRAS, which promotes survival signaling by engaging various downstream protein kinases. Targeting KRAS itself has proven to be hard, thus our strategy here is to target KRAS downstream effectors. Nevertheless, targeting one downstream pathway usually leads to compensatory activation of interconnected survival pathways. In this study, we are targeting PI3K and mTOR pathways using the novel dual PI3K/mTOR inhibitor VS-5584. We confirmed that PI3K/mTOR dual inhibitor VS-5584 causes overactivation of ERK. Furthermore, we demonstrated that ERK-selective inhibitor SCH772984 or BVD-523 enhances VS-5584-induced cell death in PDAC cell lines and showed promising antitumor activity in an HPAC xenograft mouse model; on day 29, combined treatment resulted in 80% tumor growth inhibition.

It has been reported that MEK inhibitors, U0126 and PD0325901, abrogate BEZ235-induced (a PI3K/mTOR dual inhibitor) ERK activation in KRAS mutant pancreatic cancer cell lines [21]. Similarly, we found that in KRAS mutant cell line HPAC, the MEK inhibitor GSK1120212 abrogated VS-5584-induced ERK activation and strongly enhanced VS-5584-induced cell death (Figure 3). In contrast, we found that in a KRAS wild-type cell line, BxPC-3, GSK1120212 abrogated VS-5584-induced ERK activation, but it did not robustly enhance VS-5584-induced cell death (Figure 3). Unlike the MEK inhibitor, the ERK-selective inhibitor SCH772984 did enhance VS-5584-induced cell death in BxPC-3 cells, which is consistent with Hayes and colleagues who reported that SCH772984-sensitive pancreatic cancer cell lines (all KRAS mutants) tended to be resistant to the MEK inhibitor selumetinib [26]. They also reported that treatment of PDAC cell lines with lower concentrations of SCH772984 (< 1 μM) and shorter times (< 24 h) decreased p-ERK levels, as would be expected from an ERK-selective inhibitor. However, with higher concentrations (up to 4.8 μM) and/or longer treatment time (up to 72 h) they found that p-ERK levels increased. As shown in Figures 4A and 4B and 6G&H, our results corroborate their findings. As Hayes et al. reported, this restoration of p-ERK was likely due to the loss of ERK-mediated feedback inhibition of the KRAS-RAF-MEK pathway [26]. ERK-selective inhibitor BVD-523 significantly enhanced VS-5584-induced cell death in HPAC cells, it caused increase of p-ERK and combination with VS-5584 did not reduce VS-5584-induced p-ERK levels; however, there was a substantial decrease of total ERK levels. Taken together, our results suggest that ERK has other functions (potentially independent of phosphorylation) which are important factors for cell death induced by combined VS-5584 and ERK inhibition.

How dual PI3K/mTOR inhibitor VS-5584 causes activation of ERK remains unknown. ERK activation was detected at VS-5584 concentrations which did not decrease p-AKT but did decrease p-S6 (downstream of mTOR complex 1), suggesting that inhibition of mTOR complex 1 caused activation of ERK, which is in agreement with Carracedo and colleagues [27]. Though, at higher concentrations of VS-5584, inhibition of both PI3K and mTOR was evident, as determined by decreased p-AKT and p-S6, suggesting that other factors may be involved. As Soares and colleagues proposed [21], dual inhibition of PI3K/mTOR may cause feedback activation of ERK through a PI3K-independent mechanism.

VS-5584 has been shown to preferentially diminish breast and ovarian cancer stem cells [28]. While it would be important to investigate the effectiveness of VS-5584 in combination with SCH772984 against pancreatic cancer stem cells, it is beyond the scope of this paper. In conclusion, our results show that VS-5584 treatment causes activation of ERK, which can be overcome by using ERK-selective inhibitor SCH772984 or BVD-523 in PDAC cells. In addition, we show that VS-5584 and SCH772984 synergize in PDAC cell lines and in a PDAC xenograft model. The data presented provides compelling support for the clinical development of VS-5584 in combination with an ERK-selective inhibitor in the treatment of pancreatic cancer.

## MATERIALS AND METHODS

### Drugs

VS-5584, GSK1120212, SCH772984, and BVD-523 were purchased from Selleck Chemicals (Houston, TX, USA).

### Cell culture

The AsPC-1 (KRAS mutant), BxPC-3 (KRAS wild-type), CFPAC-1 (KRAS mutant), HPAC (KRAS mutant), MIAPaCa-2 (KRAS mutant), and PANC-1 (KRAS mutant) human PDAC cell lines were purchased from the American Type Culture Collection (ATCC; Manassas, VA, USA) and cultured as previously described [29]. The cell lines were authenticated in 2013 by the University of Arizona Genetics Core Facility (Tucson, AZ, USA) using the Promega PowerPlex16HS assay. The cell lines were tested for the presence of mycoplasma on a monthly basis using the PCR based method described by Uphoff and Drexler [30].

### *In Vitro* cytotoxicity assays

*In vitro* cytotoxicities of VS-5584 and SCH772984 or BVD-523, alone or in combination, in the PDAC cell lines were measured by MTT (3-[4,5-dimethyl-thiazol-2-yl]-2,5-diphenyltetrazolium-bromide, Sigma-Aldrich, St Louis, MO, USA) assay, as previously described [31–33]. IC_50_ values were calculated as drug concentrations necessary to inhibit 50% growth compared to vehicle control treated cells. The extent and direction of antitumor interactions were determined by standard isobologram analyses, as described previously [31, 34].

### Cell death and cell cycle progression

PDAC cells were treated with the indicated drugs for up to 48 h. DNA content was determined by propidium iodide (PI) staining and flow cytometry analysis using a FACScan flow cytometer (Becton Dickinson, San Jose, CA, USA), as previously described [34]. Cell cycle analysis was performed using Multicycle software (Phoenix Flow Systems, Inc., San Diego, CA, USA). Cell death is expressed as the percent of cells with sub-G1 DNA content. Histograms were created using FlowJo v7.6.5 (Tree Star, Ashland, OR, USA). The extent and direction of antileukemic interaction for VS-5584 and BVD-523 was determined by calculating the combination index (CI) values using CompuSyn software (Combosyn Inc., Paramus, NJ). CI < 1, CI = 1, and CI > 1 indicate synergistic, additive, and antagonistic effects, respectively [31, 35].

### Western blot analysis

Whole cell lysates were prepared by sonication in 10 mM Tris-Cl, pH 7.0, containing 0.5% SDS, protease inhibitors, and phosphatase inhibitors (Roche Diagnostics, Indianapolis, IN, USA). Whole cell lysates were subjected to SDS-polyacrylamide gel electrophoresis, electrophoretically transferred onto polyvinylidene difluoride (PVDF) membranes (Thermo Fisher Inc., Rockford, IL, USA) and immunoblotted with anti-PARP, -Mcl-1, -Bcl-2, -Bcl-xL, -Bax, -actin, -Bad, -ERK (Proteintech, Chicago, IL, USA), -p-AKT (T308), -p-AKT (S473) (Affinity Biosciences, Changzhou, Jiangsu Province, China), -Bim, -pS6 (Cell Signaling Technologies, Danvers, MA, USA), -p-ERK, or -AKT (Abcam, Cambridge, MA, USA), as previously described [31, 33, 36, 37]. Immunoreactive proteins were visualized using the Odyssey Infrared Imaging System (Li-Cor, Lincoln, NE, USA), as described by the manufacturer. Western blots were repeated three times; one representative blot is shown.

### Transwell migration assay

BxPC-3 and HPAC cells were cultured in serum-free medium for 24 h. Cells at 80% confluence were trypsinized. Trypsin was neutralized with medium containing 5% bovine serum albumin (BSA). The cells were resuspended in culture medium containing 0.2% BSA and were seeded (1x 10^5^ cells/well) into 8 micron transwell chambers and then VS-5584 (final concentration 0.25 μM) and SCH772984 (final concentration 0.25 μM), alone or in combination, were added to bring the final volume to 100 μL. The chambers were inserted into a 24-well plate containing 650 μL medium with 5% FBS and corresponding concentrations of VS-5584 and SCH772984, alone or in combination. The cells were incubated for 24 h, and then the cells on the surface of the top chamber were removed using cotton-tipped applicators. The cells on the bottom surface of the chamber were fixed with methanol, stained with crystal violet, and then washed twice with PBS. Images were taken using a light microscope with a 10 x objective lens. Stained cells were then eluted using 33% acetic acid and absorbance was determined at 570 nm. Results were obtained from three independent experiments. The cell migration rates, compared to control, are shown as mean ± SEM.

### Establishment of a mouse PDAC xenograft model

Female BALB/c nude mice (18–22 g) were purchased from Vital River Laboratories (Beijing, China). The animal study was conducted following internationally recognized guidelines and was approved by the Animal Research Committee of Norman Bethune College of Medicine, Jilin University. The HPAC xenograft model was generated as previously described [29, 38]. Briefly, HPAC cells were adjusted to a density of 2 × 10^7^ cells/mL with 50% v/v matrigel (BD Biosciences, San Jose, CA, USA) and inoculated subcutaneously in the right side axillae (0.1 mL/mouse). Once the tumor diameter reached approximately 0.5 cm it was isolated, cut into small pieces (1 mm in diameter) and then subcutaneously implanted unilaterally along the right flanks of mice. When the xenografts reached a volume of 104.3 ± 13.4 mm^3^, the mice were randomized into four groups (7 animals per group, the mean tumor volumes ± SD were 104.1 ± 16.7, 104.4 ± 12.1, 104.7 ± 15.3, and 103.7 ± 12.1 mm^3^ for the vehicle control, VS-5584, SCH772984, and combination groups, respectively) and chronically treated on a daily schedule for four weeks (QDx28days) as follows: (i) vehicle control for both drugs at 0.1 mL/injection, (ii) 8.4 mg/kg VS-5584 by p.o., (iii) 25 mg/kg SCH772984 by ip, or (iv) 8.4 mg/kg VS-5584 by p.o. and 25 mg/kg SCH772984 by ip. Tumor diameters were measured with a caliper daily. Tumor volume was calculated according to the following formula: m1^2^ x m2 × 0.5236 (m1: short diameter; m2: long diameter). The mice were sacrificed on day 29, 1-day post last drug treatment (first drug treatment day was designated day 1).

### Hematoxylin & eosin (H&E) and immunohistochemical staining

On day 29, tumors from 3 mice in each treatment group were excised for H&E staining, and Ki-67 and CD34 immunohistochemical staining, as previously described [29, 38]. Major organs from the same mice were also harvested for H&E staining. The slides were analyzed using a microscope and brown staining was scored using Image-Pro Plus 6.0 (Media Cybernetics, Inc., Bethesda, MD, USA).

### Statistical analysis

Differences in cell death and cell migration among treatment groups (vehicle control, VS-5584, SCH77298 or BVD-523, and the combination) were compared using one-way ANOVA with Bonferroni post hoc test. A linear mixed effects model [39]; estimating an interaction fixed effect between time and treatment, an animal-specific random effect, a different variance within each experimental group and a first order autocorrelation structure for observations on the same animal; was constructed to determine the treatment effect on the rate of tumor growth using the nlme package in R [40]. %T/C was calculated using the formula: T/C x 100; determined on selected day post all treatment (day 29) when control tumors were still in exponential growth phase, using the mean treated (T) and control (C) tumor values from each group. All other statistical analyses were performed with GraphPad Prism 5.0. Error bars represent ± SEM. The level of significance was set at *p* < 0.05.

## SUPPLEMENTARY MATERIALS FIGURE



## Abbreviations

PDAC: pancreatic ductal adenocarcinoma
CI: combination index
PI: propidium iodide
H&E: hematoxylin & eosin
p.o.: oral gavage
ip: intraperitoneal injection
T: mean treated tumor volume
C: control treated tumor volume.
