# Supplementary material for: Targeting ERK enhances the cytotoxic effect of the novel PI3K and mTOR dual inhibitor VS-5584 in preclinical models of pancreatic cancer
Source: Oncotarget. 2017 May 15;8(27):44295–311. doi: 10.18632/oncotarget.17869 (PMC5546481; doi:10.18632/oncotarget.17869)
Supplement: Supplementary file 1 [file oncotarget-08-44295-s001.pdf]

Targeting ERK enhances the cytotoxic effect of the novel PI3K and mTOR dual inhibitor VS-5584 in preclinical models of pancreatic cancer

Supplementary Materials

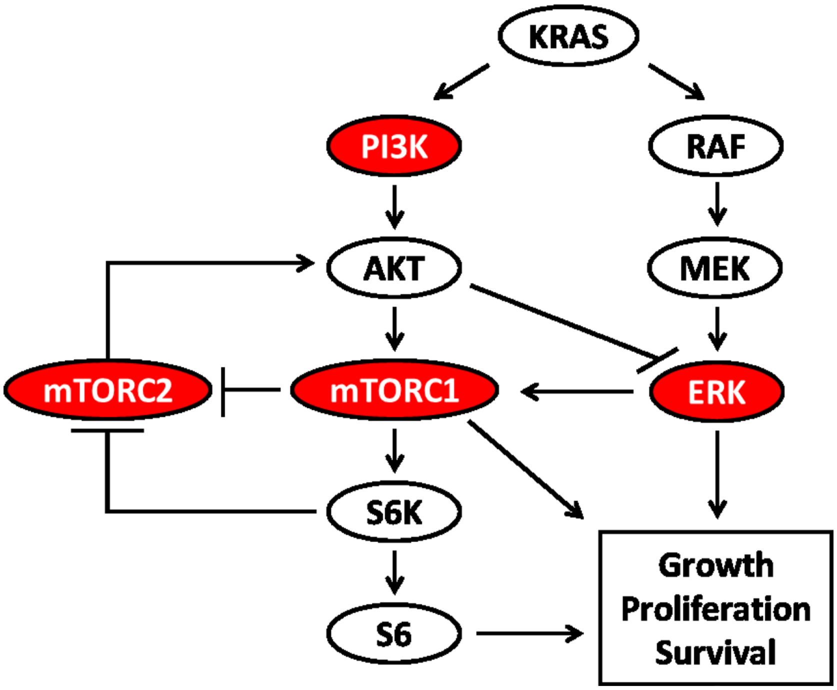

Supplementary Figure 1: Simplified schematic of the cross-talk between the PI3K/mTOR and MEK/ERK pathways.
